# Editorial: Methodological considerations in sports science, technology and engineering

**DOI:** 10.3389/fspor.2023.1294412

**Published:** 2023-10-04

**Authors:** Alan Godfrey, Samuel Stuart, Ian C. Kenny, Thomas M. Comyns

**Affiliations:** ^1^ Department of Computer and Information Sciences, Northumbria University, Newcastle upon Tyne, United Kingdom; ^2^ Department of Sport, Exercise and Rehabilitation, Northumbria University, Newcastle upon Tyne, United Kingdom; ^3^ Department of Neurology, Oregon Health & Science University, Portland, OR, United States; ^4^ Department of Physical Education and Sport Sciences, University of Limerick, Limerick, Ireland; ^5^ Health Research Institute, University of Limerick, Limerick, Ireland; ^6^ Sport and Human Performances Research Centre, University of Limerick, Limerick, Ireland

**Keywords:** wearable, sport performance, digitization in sport, sports medicine, technology

**Editorial on the Research Topic** Methodological considerations in sports science, technology and engineering

## Introduction

The use of digital technology in sport science has been on the rise in recent years (1–4). This is due to the development of new and innovative technologies that can be used to e.g., measure and analyse athletic performance such as use of inertial wearables in a range of sports (5–9). Some of the most common technologies used in sport science include wearable devices, motion capture systems and data analytics, including use of machine learning (10). Those technologies are used for a variety of purposes in sport science, including improving athletic performance, reducing injury frequency, rehabilitating injuries and/or analysing tactical performance. However, the use of novel technology in sport science is not without its challenges. Notable challenges include the cost of those technologies and the need for specialized expertise to use these technologies effectively. Moreover, there is often a lack of research on reliability and validity of new emerging technologies whereby published research often lags technological development. Despite the challenges, use of contemporary technology in sport science is a rapidly growing field. This is due to the many benefits that these technologies can offer athletes, coaches, and sports scientists.

The future of sport science is likely to be even more technology driven. As new technologies are developed, they will be used to e.g., further improve athletic performance and reduce injuries. By carefully considering the methodological aspects involved in sport science, technology, and engineering research, researchers can help to ensure that their findings are reliable, valid, and generalizable i.e., fit for purpose. This will ultimately help to improve our understanding of the factors that influence human performance and to develop new technologies and interventions that can enhance athletic performance. This series aims to highlight the latest methods, experimental techniques used to investigate fundamental questions in sport science, technology and engineering research, from the use of technology to engineering design. This Research Topic includes research which help advance science. This special issue encompasses several articles exploring integrating new technologies and statistical models in sport and exercise science research.

## Articles

The first (Young et al.) examines the validity of a low-cost, foot-mounted inertial measurement unit (IMU) methodology for assessing running gait. The methodology uses a zero-crossing (ZC) algorithm to identify the initial contact of the foot with the ground, and then uses this information to extract features such as foot strike location, pronation severity, and ground contact time. The authors compared the performance of the ZC-based methodology to that of Vicon 3D motion tracking data in a group of 20 participants who ran at a range of speeds from 8 to 16 km/h. They found that the ZC-based methodology was able to extract pronation, foot strike location, and ground contact time with good to excellent agreement with the Vicon data for a range of speeds between 8 and 12 km/h. However, the performance of the ZC-based methodology began to deteriorate at higher speeds (14 km/h+), suggesting that other features and approaches may be more suitable for faster running and sprinting tasks.

The second (Reichert et al.) investigated the validity and reliability of a new method for measuring isometric horizontal strength in game sport athletes. The study involved 119 athletes from different game sports, including American football, handball, and basketball. The isometric horizontal strength was measured in three game-like standing positions: upright, slightly leaning forward, and clearly leaning forward. Each position was tested in three weight-shift conditions: 80% of body weight on the left leg, 50/50% on both legs, and 80% on the right leg. Handgrip strength was also measured on both sides. The results showed that the new method for measuring isometric horizontal strength was reliable, with high levels of within-test reliability (ICC > 0.90) and test-retest reliability (*r* > 0.77). The results also showed that handgrip strength was a significant predictor of upper-body horizontal strength in female athletes but not in male athletes. The number of years played at the top level was also a significant predictor of upper-body horizontal strength. The authors concluded that the new method for measuring isometric horizontal strength is a valid and reliable tool for assessing performance-relevant upper-body horizontal strength in game sport athletes. They also suggested that handgrip strength and the number of years played at the top level could be used as predictors of upper-body horizontal strength in game sport athletes. In addition to the findings mentioned above, the study also found that the isometric horizontal strength was greater in the upright position than in the slightly forward leaning position, and greater in the slightly forward leaning position than in the clearly leaning forward position. This suggests that the isometric horizontal strength is influenced by the postural position of the athlete.

The third (Ranaweera et al.) presents a case study on how a professional Rugby Union club in England used digital technologies to optimize the information flows necessary to manage their athletes. The club's High-Performance Unit (HPU) was responsible for the health and fitness of the players, and they identified that the quality of the information they were receiving was not good enough to make informed decisions about player management. The club used a Business Process Management (BPM) approach to redesign the information flow, and they also used the Lean Startup framework to develop and test new digital solutions. The results of the study showed that the new information flow was significantly more efficient and effective than the old one. There were also positive improvements in the quality of the information, with major improvements in accessibility. The study's findings suggest that digital technologies can be used to improve the information flows necessary to manage professional athletes. This is important because good information is essential for making informed decisions about player health, fitness, and performance. The methods employed in the study could be used by other sporting organizations to improve their own information flows. The study is a valuable contribution to the literature on upper-body strength in game sport athletes. The findings of the study can be used to help coaches and athletes develop training programs that target upper-body horizontal strength.

Lastly, Mai et al. investigated the effect of unanticipated fake-and-cut manoeuvres on knee abduction moments (KAMs) in female handball players. KAMs are a risk factor for anterior cruciate ligament (ACL) injuries, which are common in sports that involve cutting, such as handball. The study included >50 female handball players who performed three different fake-and-cut tasks. Results show that the KAMs were highest in the pre-planned cut with a static defender, and lowest in the simple pre-planned cut. The authors interpreted their findings to suggest that female handball players have developed an automated sport-specific cutting technique that is utilized in both pre-planned and unanticipated fake-and-cut tasks. This technique is likely designed to minimize KAMs and reduce the risk of ACL injury. The findings of this study have implications for ACL injury prevention and risk screening. The results suggest that unanticipated fake-and-cut manoeuvres do not pose a greater risk of ACL injury than pre-planned cutting manoeuvres. Therefore, screening athletes for ACL injury risk should not focus solely on unanticipated cutting manoeuvres. Instead, it is important to assess the athlete's overall cutting technique and identify any risk factors, such as poor alignment or muscle weakness. In addition, the findings of this study suggest that sport-specific training programs should focus on developing an automated cutting technique that minimizes KAMs. This can be done by teaching athletes how to properly align their body and use their muscles during cutting manoeuvres.

## Conclusions

The papers that were included in the Research Topic emphasize various methodological, technical, and practical issues that need to be considered when incorporating new technology and statistical models into sport and exercise science research. Insights regarding the advantages, drawbacks, and possible applications across a range of sporting topics are presented: novel inertia-based wearables, strength training, player management, and injury risk through detailed analysis. Researchers may improve data collecting, processing, validation processes and interpretation by considering these factors presented throughout this Research Topic, which will inevitably result in improvements to sports and exercise science research and practical applications.

## Author contribution

AG: Writing – original draft. SS: Writing – review & editing. IK: Writing– review & editing. TC: Writing – review & editing.
